# Urine cortisol‐creatinine and protein‐creatinine ratios in urine samples from healthy dogs collected at home and in hospital

**DOI:** 10.1111/jvim.15735

**Published:** 2020-02-13

**Authors:** Lindsey E. Citron, Nicole M. Weinstein, Meryl P. Littman, Jonathan D. Foster

**Affiliations:** ^1^ Friendship Hospital for Animals Washington District of Columbia; ^2^ University of Pennsylvania School of Veterinary Medicine Philadelphia Pennsylvania

**Keywords:** hypercortisolemia, hypercortisoluria, proteinuria, stress

## Abstract

**Background:**

Recently, urine protein:creatinine ratios (UPC) were shown to be lower in urine samples from dogs collected at home (AH) as compared to those collected in hospital (IH). Stress‐inducing procedures and travel to the hospital have been hypothesized to cause prerenal proteinuria.

**Objectives:**

Evaluate patient stress using urine cortisol:creatinine ratios (UCCr) and correlate UCCr to UPC in urine samples obtained AH and IH.

**Animals:**

Thirty‐six healthy, client‐owned dogs.

**Methods:**

Prospective, non‐masked study. Two voided urine samples were obtained (AH and IH). Complete urinalysis as well as UPC and UCCr were performed. Clients graded their dogs' stress level AH, in transport, and IH.

**Results:**

The UCCr was significantly higher in IH samples than in AH samples (*P* < .0001), but UPC was not significantly different between AH and IH urine samples (*P* = .14). In all samples and in both collection settings, UCCr was not significantly correlated with UPC. Travel time and time IH were not correlated with change in UCCr or UPC. In 8 dogs with borderline or overt proteinuria, no significant difference was found in UPC between settings, but UCCr was significantly higher in IH samples.

**Conclusions and Clinical Importance:**

The UPC was not higher when measured in urine samples collected IH compared to AH. Dogs had higher UCCr IH, but UCCr was not associated with UPC. Stress, as estimated by UCCr, did not affect proteinuria. Further evidence is needed to support the claim that stress may result in proteinuria in healthy dogs.

**Abbreviations**
AH: at home
IRIS: International Renal Interest Society
IH: in hospital
UCCr: urine cortisol:creatinine ratio
UPC: urine protein:creatinine ratio

## 1 INTRODUCTION

The method and timing of urine collection may influence urinalysis and other urine test results.^1^, ^2^ Voided urine samples may be obtained by the client at home (AH) or samples may be collected during the patient's evaluation in hospital (IH). Urinalyses are routinely used to evaluate kidney function, including semiquantitative and quantitative measurement of urine protein concentration. Proteinuria can be extrarenal or renal in origin, and can be due to prerenal, renal, or postrenal processes. Pathologic renal proteinuria often is the most severe type of proteinuria, especially when the cause is altered glomerular permselectivity. In particular, pathologic renal proteinuria is considered a marker for increased risk for progression of kidney disease and prompts the need for intervention by practitioners. However, other nonrenal factors, such as stress, have been hypothesized to cause transient, nonpathologic prerenal proteinuria in dogs.^3^

Several publications have demonstrated that emotional stress may result in transient proteinuria in humans. Altered urine concentrations of neurohormones such as endothelin‐1 and prostaglandins have been observed after stressful stimuli, which may affect glomerular permselectivity.^4^ Altered permeability of the glomerular barrier typically results in the appearance of high molecular weight proteins, such as albumin, in the urine. Several qualitative metrics of emotional stress have been observed to correlate with urine total protein concentration and with both urine protein:creatinine (UPC) and albumin:creatinine ratios in people.^5^, ^6^ Stress also was associated with increased urine concentration of malondialdehyde, a marker of lipid oxidation, suggesting oxidative stress may play a role in the etiology of proteinuria.^5^ Although no studies have determined that stress causes proteinuria in dogs, some veterinarians believe stress to be a cause of proteinuria.^7^, ^8^

Serum and urine cortisol concentrations can be used to assess stress in dogs.^9^, ^10^ The IH setting can induce a stress response in dogs, as previously indicated by increased serum cortisol concentrations compared to dogs that stayed outside the hospital.^11^ Similarly, veterinary care causes stress in dogs and increases their urine cortisol:creatinine ratio (UCCr) while IH compared to AH.^12^ Both naturally occurring and iatrogenic hypercortisolemia have resulted in increased UPC in dogs.^13^, ^14^, ^15^, ^16^, ^17^

A recent study found that UPC ratios were higher when urine was collected IH compared to AH in dogs with positive urine dipstick protein results.^3^ Stress was a hypothesis provided by the authors as to the cause of the higher UPC observed IH, but no assessment of stress was performed in that study.

Our primary objective was to investigate if higher UPC observed in IH samples is caused by stress, as assessed by the UCCr and to test the hypothesis that urine from dogs experiencing higher stress as a result of an IH medical appointment will have increased UCCr and UPC when compared to results from samples obtained from the dogs AH. A secondary objective was to evaluate the correlation between UCCr and UPC. Lastly, we examined whether duration of time IH, or transit time to the hospital causes significant changes in UPC and UCCr.

## 2 MATERIALS AND METHODS

Our study was a prospective, non‐masked pilot study. Forty‐three client‐ and staff‐owned dogs were eligible for study screening between May 2016 and September 2016. Dogs with preexisting or suspected conditions that could increase the UCCr for non‐stress‐related causes (eg, hyperadrenocorticism, dogs currently receiving corticosteroids, or hypoadrenocorticism) as well as dogs with clinically relevant systemic disease (anorexia, lethargy, fever, acute diarrhea, vomiting, malaise, as well as chronic renal disease) were excluded. Dogs were assessed to be free of systemic disease based on historical findings, physical examination findings, and any diagnostic tests performed at the discretion of the attending clinician. Participants were recruited from the Small Animal Ophthalmology and Community Practice services at the Matthew J Ryan Veterinary Hospital of the University of Pennsylvania by phone call at least 1 day before their appointment. Clients who gave verbal consent for enrollment in the study were asked to collect a mid‐stream voided urine sample in a dry clean plastic cup or bag on the morning of their appointment (within 12 hours of the appointment time) and to store the sample in a refrigerator until leaving for the hospital. Clients were asked to use cups that had never been in the dishwasher to avoid detergents that could interfere with laboratory testing. Dogs were enrolled regardless of the presence of proteinuria on urinalysis examination.

Upon arrival at the hospital, clients submitted the urine samples to investigators and samples were time stamped. Clients were given a client consent form to sign, as well as a short survey to complete, which inquired about travel time from their home to the hospital. The survey also asked clients to grade their dog's stress level from 0 to 10 (0 being nonstressed and 10 being severely stressed [the most stressed their pet could be]) during the AH urine collection, the car ride to their appointment, and at the time of survey administration in the hospital lobby. Study dogs completed their scheduled appointments and were taken to an enclosed space to obtain an additional voided urine sample. Dogs that required cystocentesis were excluded from the study. Hospital sample collection time was noted to determine the total duration of time IH, and additional data (age, weight, breed, reason for visit) were collected from the medical history and examination forms. A minimum urine volume of 3 mL was required for study eligibility. Dogs qualified for enrollment regardless of whether or not they were positive for proteinuria on a urine dipstick analysis.

Urine protein:creatinine ratios were interpreted according to the American College of Veterinary Internal Medicine and International Renal Interest Society (IRIS) guidelines on proteinuria: UPC <0.2 = normal, 0.2‐0.5 = borderline, and >0.5 = overtly proteinuric.^18^, ^19^ These standards were used to evaluate discrepancies among UPC results and assess clinical relevance.

Urine protein and creatinine concentrations were measured using pyrogallol red‐molybdate and creatinase methods, respectively, on an automated chemistry analyzer (Ortho Clinical Vitros 4600) within 24 hours of collection. If submitted after hours, samples were refrigerated at 4°C until testing was performed the next day. Urine cortisol concentration was measured at an external reference laboratory using a chemiluminescent bead‐based assay (Siemens Immulite 2000). A conventional urinalysis also was performed and included evaluation of macroscopic characteristics, specific gravity, chemical analysis by dipstick, and microscopic sediment examination.

### 2.1 Statistical methods

The small sample size was presumed not to be normally distributed, and therefore nonparametric tests were utilized. A Wilcoxon signed rank test was used to evaluate AH versus IH variables including UPC and UCCr. Owner perceived stress scores for their dogs AH, during travel, and IH were analyzed using Friedman's test. Spearman's analysis was used to assess the strength of correlation between UPC and UCCr as well as the effect of travel time and duration of visit on UPC. A *P* value <.05 was considered significant. A commercial statistical software program was used for all analyses (Prism 7 for Mac, GraphPad Software Inc, La Jolla, California).

## 3 RESULTS

**Figure 1:**
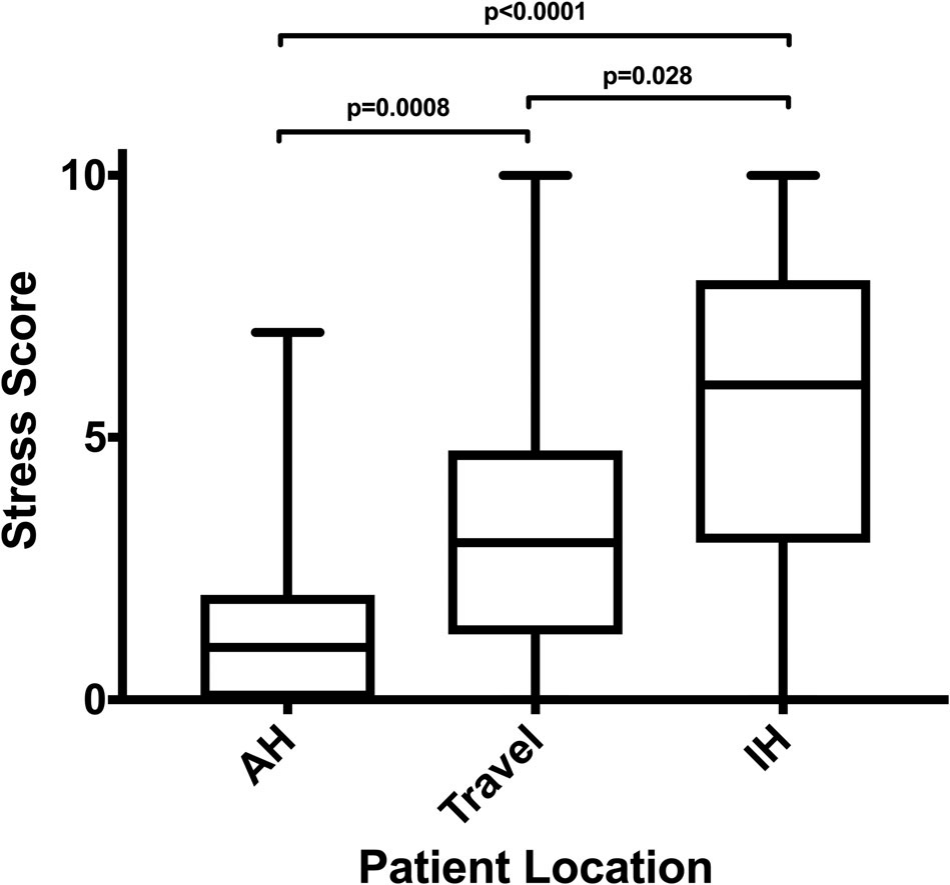
Owner perceived stress scores while their dog is at home (AH), traveling to the hospital, or in hospital (IH)

Thirty‐six dogs were enrolled in the study: 33 dogs presented for annual physical examinations or vaccinations to the primary care service and 3 dogs presented for ophthalmologic reevaluations. The initial aim of the study was to enroll 40 dogs, but enrollment was limited to 36 because of funding limitations. Mixed breed dogs were most common (13), followed by German Shepherds (3) and Chihuahuas (2). Median body weight was 22.15 kg (range, 2.1‐71 kg), and median age was 6 years (range, 3‐14 years). There were 21 neutered male, 8 spayed female, 5 intact male, and 2 intact female dogs. Dogs were not selected based on sex or breed. The median duration of time spent in the hospital was 100 minutes (range, 40‐315 minutes), and the median client travel time from home to the hospital was 30 minutes (range, 5‐210 minutes). The owners' perception of their dogs' stress level was significantly different in each environment (*P* < .0001). Stress score was highest IH (median, 6; range, 0‐10), compared to in transit (median, 3.5; range, 0‐10) and AH (median, 1; range, 0‐7; Figure 1). Eight of the 36 dogs (22%) were at least borderline proteinuric with 5 of those 8 dogs having at least 1 measurement >.5 and consistent with overt proteinuria. One of these proteinuric patients was an intact male.

No significant difference was identified in UPC between urine samples obtained AH versus IH, which also was true in the subpopulations of dogs that were borderline (UPC >0.2) or overtly proteinuric (UPC >0.5) in at least 1 sample (Table 1).

**Table 1:** Urine protein:creatinine ratios (UPC) in at‐home versus in‐hospital samples; *P*‐value for Friedman test comparing groups

UPC		At home	In hospital	*P* value
All dogs (n = 36)	Median	0.02	0.03	.14
	Range	0.01‐2.64	0.01‐2.65	
Dogs with UPC >0.2 (n = 8)	Median	0.535	0.695	.25
	Range	0.02‐2.87	0.11‐2.64	

The UCCr results were significantly higher for IH samples compared to those collected AH (*P* = .0001; Table 2), where 30 of 36 (83.3%) dogs had a higher UCCr in the sample obtained IH compared to AH. In 7 of 36 (19.4%) IH samples, the UCCr was higher than the normal reference interval (<34). Within the subpopulations of borderline and overtly proteinuric dogs, the UCCr remained significantly higher for IH‐collected samples compared to those collected AH (*P* = .03).

**Table 2:** Urine cortisol:creatinine ratios (UCCr) in at‐home versus in‐hospital samples; *P*‐value for Friedman test comparing groups

UCCr		At home	In hospital	*P* value
All dogs (n = 36)	Median	17.5	20.5	.0001
	Range	1‐41	10‐189	
Dogs with UPC > .2 (n = 8)	Median	13	17	.031
	Range	1–41	11‐159	

The UPC was not significantly correlated with UCCr (r_s_ = .018; *P* = .88,). This was true for all urine samples as well as when only evaluating urine collected AH (r_s_ = −.075; *P* = .66) or IH (r_s_ = .02; *P* = .91). Travel time and time spent IH were not associated with changes in UPC (*P* = .14 and *P* = .45, respectively) or UCCr (*P* = .50 and *P* = .73, respectively). The observed change in AH and IH UPC was not associated with the change between AH and IH UCCr (*P* = .14).

## 4 DISCUSSION

In our study, UCCr was higher in samples collected from dogs at a veterinary hospital compared to UCCr in samples collected AH, before transport. This finding is similar to those of previous studies where veterinary care and setting were shown to increase overall stress level.^10^, ^11^, ^12^ Despite the increase in UCCr suggesting an increase in stress, dogs did not experience a concurrent significant increase in UPC, regardless of the initial UPC result. The similar findings in UPC in AH and IH environments differ from results of previous studies.^3^, ^20^ In our study, in which urine dipstick protein results did not determine study participation, a small proportion of dogs were either borderline or overtly proteinuric. Only 8 of 36 dogs (22%) had an increased UPC result at any time point and only 5 dogs (14%) had a UPC >0.5 at any time point. None of the 8 dogs had an active sediment on either the AH or IH samples, and the median urine specific gravity was 1.020 (range, 1.005‐1.043) and 1.009 (range, 1.005‐1.030), respectively, for AH and IH samples. The cause of proteinuria in this subset was unknown, as none of these dogs were being evaluated specifically for proteinuria at the time of enrollment. One of the proteinuric dogs was an intact male; studies have shown intact males to have higher urine dipstick measurement of proteinuria but the effect on UPC is not known.^21^ Proteinuric dogs did have higher UCCR in samples collected IH, but their UPC was not significantly different. In an earlier study, 50% of the dogs had UPC >0.5 at either time point.^3^ Within that group of dogs, UPC was increased in urine samples collected IH compared to those collected AH. In dogs with UPC <0.5, UPC did not increase related to AH or IH setting, similar to our results. It was speculated, in the previous study, that the increased UPC observed IH could be a result of stress, but no assessment of stress was included in that study, nor were timing and method of urine collection standardized. Our study showed dogs experienced stress while at a veterinary hospital, as indicated by significantly increased UCCr, but no correlation between UCCr and UPC was identified.

In people, the mechanism by which stress causes proteinuria is not fully understood. Proposed mechanisms include alterations in glomerular capillary permeability, oxidative damage, and glomerular hypertension.^4^, ^6^, ^15^ Studies have shown that >50% of dogs with naturally occurring hypercortisolemia have proteinuria, with most affected dogs having a UPC <5.0.^13^, ^14^, ^16^, ^17^ The protracted nature of hyperadrenocorticism may result in pathophysiological changes, such as chronic systemic arterial hypertension, glomerulosclerosis, and hypercoagulability, which may affect glomerular permeability.^22^ These changes may not develop during the comparatively shorter duration of stress and hypercortisolemia associated with travel to a veterinary hospital and a brief hospital stay. It is possible that existing glomerulotubular pathology may predispose dogs to stress‐related proteinuria that is not observed in dogs with normal kidney structure and function. Further evaluation should be performed in a larger population of known proteinuric dogs to determine if any different observations are encountered and if stress is correlated with any observed changes. Additionally, although UCCr currently is regarded as a reliable measure of stress in dogs, it is possible that other serum or urinary biomarkers could better quantify stress levels in dogs.^10^

Identification of proteinuria in azotemic and non‐azotemic dogs will influence treatment recommendations, based on the IRIS staging protocol. Consistency when monitoring a proteinuric dog is recommended.^18^, ^19^ This includes minimizing the analytical variability such as using the same instrument and laboratory for repeated measurements as well as limiting preanalytical contributions such as time between specimen collection and analysis, the potential influence of sample collection timing, and variation between single and pooled specimens.^23^, ^24^, ^25^

Twenty‐four‐hour urine collection for protein quantification is considered the gold standard method for assessing proteinuria.^20^ Although the UPC does correlate with 24‐hour urine protein excretion, variability can occur in spot UPC results.^26^, ^27^ The nature of our study necessitated the use of single voided urine samples, rather than pooled or averaged samples to measure UPC. Pooled samples are more cost‐effective but do not necessarily provide a diagnostic advantage over a single urine sample when the degree of proteinuria is low to low‐moderate.^20^, ^28^ Known analytical variability coupled with biologic variation can influence results even in samples with a low degree of proteinuria. These changes, although not necessarily clinically relevant, may be mitigated using pooled samples and appropriate specimen handling.^20^, ^25^, ^28^

Further evidence is aid to support the suggestion that stress leads to abnormal urinary protein excretion and increased UPC in dogs. The effects of timing of urine collection on UPC results may be dependent on the population, and further studies are warranted in proteinuric dogs. In non‐proteinuric dogs, IH urine collection does not obviously result in proteinuria based on UPC results, which is a repeated finding in the current and previous studies.^3^ Given the current study results, proteinuria confirmed by UPC measurement in an IH‐obtained sample should therefore not be discounted. Although data from our study did not find the timing of urine sample collection to influence UPC results, the effects of timing may, at least based on the results of previous studies, influence the magnitude of increase in the UPC in dogs already known to be proteinuric.^3^, ^20^ In non‐proteinuric dogs, urine samples collected IH may yield similar results to those of samples collected AH, which would allow clinicians to appropriately quantify and evaluate the magnitude of proteinuria identified in voided IH urine samples. One limitation of our study was the subjective nature of the numerical stress scores reported by the owners. Although the owners' perception of their dogs' stress level was significantly higher for IH than for AH samples, this metric has not been studies. Another limitation of our study is the low number of overtly proteinuric dogs that were enrolled. Because urine protein results were not known before enrollment, the study population was predominantly non‐proteinuric dogs. The study population did experience a change in stress level, but this did not have any impact on UPC. Further study should be performed in proteinuric dogs to evaluate for any correlation between UCCr and UPC.

In conclusion, no difference was found in UPC in urine samples collected AH compared to IH in the current population. Dogs did have higher UCCr results in urine collected IH compared to AH, suggesting the studied population did experience stress by the completion of their examination IH. Owners' assessment of their dogs' stress level agreed with the UCCr results, suggesting that the AH environment was less stressful than travel and stay at the veterinary hospital. Potential stressors such as travel time, time spent in a potentially stressful hospital setting, and total time before urine collection were not correlated with changes in UPC or UCCr. We failed to find evidence that stress or increased UCCr causes a corresponding increase in UPC in healthy dogs. This relationship requires further investigation in dogs with glomerular proteinuria. We found that, in the case of true proteinuria in an otherwise healthy dog, stress alone may not fully explain the finding of proteinuria.

## CONFLICT OF INTEREST DECLARATION

Authors declare no conflict of interest.

## OFF‐LABEL ANTIMICROBIAL DECLARATION

Authors declare no off‐label use of antimicrobials.

## INSTITUTIONAL ANIMAL CARE AND USE COMMITTEE (IACUC) OR OTHER APPROVAL DECLARATION

This protocol has been approved by the MJR‐VHUP Privately Owned Animal Protocol Committee and the University of Pennsylvania IACUC, protocol #806051.

## HUMAN ETHICS APPROVAL DECLARATION

Authors declare human ethics approval was not needed for this study.
